# A database of two-dimensional images of footwear outsole impressions

**DOI:** 10.1016/j.dib.2020.105508

**Published:** 2020-04-14

**Authors:** Soyoung Park, Alicia Carriquiry

**Affiliations:** Iowa State University, United States

**Keywords:** Shoeprints, Image analysis, Pattern evidence, Forensic science, Statistics

## Abstract

Footwear outsole images were obtained from 150 pairs of used shoes. The motivation for constructing the database was to enable a statistical analysis of two-dimensional (2D) images of shoe outsoles, to understand within shoe (between replicate images of the same shoe) and between shoe variability, and to develop methods for the evaluation of forensic pattern evidence of shoeprints. Since we scanned the outsole of the used shoes, the images capture not only the outsole pattern design but also the marks that arise from wear and tear and that may help identify the shoe that made the impression. Each shoe in a pair was scanned five times, so that replicate images can be used to estimate within-shoe variability. In total, there are 1500 2D images in the database. The EverOS footwear scanner was used to capture the outsole of each shoe. The scanner detects the weight distribution of the person wearing the shoe when he or she steps on the scanning surface. It images the portions of the outsole that make contact with the scanning surface. The database is a useful resource for forensic scientists or for anybody else with an interest in image comparison. The database we describe, was constructed by researchers in the Center for Statistics and Applications in Forensic Evidence (CSAFE) at Iowa State University.

Specifications Table

**Table:** 

Subject	Decision Sciences; Statistics, Probability and Uncertainty
Specific subject area	Statistical image comparison with application in forensic disciplines. Data are useful for development of statistical methods to quantify the similarity between 2D images.
Type of data	Image, Table
How data were acquired	Outsoles of one hundred and fifty pairs of used shoes were scanned. We used an Everspry EverOS Footwear Outsole Scanner, which produces 2D grayscale images of the object placed on the scanning surface.
Data format	Raw Scanned outsole images have a resolution of 300 dpi and include a yellow ruler along the borders for measuring the size of the outsole.
Parameters for data collection	We obtained the 150 pairs of used shoes from volunteers. Shoe owners stepped on to the EverOS scanner, which produces a 2D image of the bottom of the shoe by detecting the distribution of wearer's weight on the surface. Each volunteer contributed 10 images: two shoes replicated five times each. The five replicate images for each shoe were obtained sequentially, but the order (left or right shoe first) was not prescribed.
Description of data collection	One hundred and fifty pairs of used shoes were imaged. The sample was a convenience sample in that the shoes belonged to friends, colleagues and office mates.
Data source location	Institution: Center for Statistics and Applications in Forensic Evidence, Iowa State University City/Town/Region: Ames, Iowa Country: U.S.A.
Data accessibility	Repository name: 2D Footwear outsole impressions hosted at Iowa State University Direct URL to data: https://doi.org/10.25380/iastate.11624073.v1

## Value of the data

• These data are among the first publicly available 2D images of footwear outsoles. There is much interest in the development of objective methods to compare a shoe to an impression found at a crime scene, yet data that would enable this type of research are scarce. The data we describe will help researchers to explore the within shoe (between replicate images of the same shoe) and between shoe variability of footwear outsoles.
• The database is useful for researchers because of three important attributes: (1) We know “ground truth” for each image, where here ground truth means that we know which shoe made which image. (2) Each outsole was imaged five times, allowing for estimation of within-shoe variability in the capture of the image. (3) The database includes a wide variety of outsole patterns, sizes, and degree of wear and tear.
• Two additional attributes that make these data useful are: (1) Images can be flipped, so from one pair of shoes we can create two right shoe images. Since both shoes are worn by the same person on the same surfaces and for the same amount of time, the differences between the true right shoe images and the flipped left shoe images can be used to investigate the distribution of randomly acquired characteristics resulting from wear and tear. (2) Several participants contributed more than one pair of shoes of the same make and model. These “repeated” shoes can be used to compare images of shoes that share class characteristics (brand, model and size) but that have potentially different unique markings.
• Forensic scientists who work on the analysis and interpretation of pattern evidence may find these data useful. In general, anyone with an interest in image analysis will be able to use the data to develop comparison algorithms, methods to extract robust features and approaches to align images and quantify the pairwise similarity between them.
• Understanding the variability within and between patterns in a collection of experimental units is important for decision-making, and in particular, in the analysis and interpretation of forensic pattern evidence.
• These data can also be used for proficiency testing of footwear examiners, or as part of training programs for forensic scientists. They can also be used as a testbed for algorithm developers. Since the EverOS scanner is widely available and reasonably priced, others can decide to expand the database by contributing additional images.

## 1 Data

The database includes 2D images of the outsole of shoes. Images were obtained from 150 pairs of used shoes (300 shoes). Each shoe was imaged five times, so the database contains 1500 2D images.

**Fig. 1:**
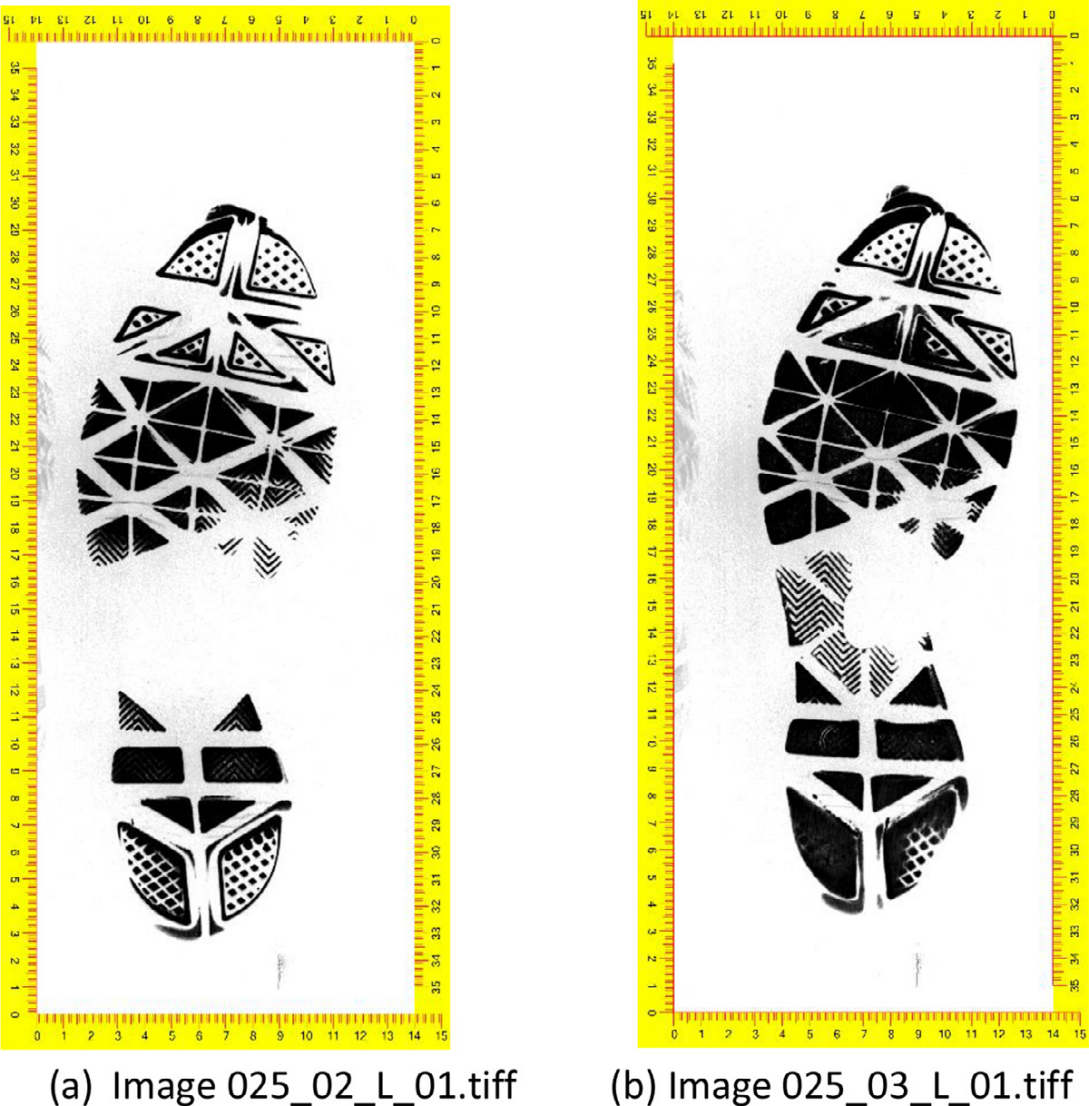
Footwear outsole images created using the EverOS footwear scanner. Fig. 1a and b show the scanned outsole of two shoes of the same brand, model, size and owner.

The images in the database contain a border with a ruler for measuring the size of the outsole. Because the shoes are used, images capture the pattern of the outsole itself as well any marks that result from wear and tear. Images are presented in grey scale. Fig. 1a and b show images of the outsole of the left shoe from two different pairs of shoes of the same brand and model owned by the same person. The outsole pattern is the same, but shoes were worn for a different amount of time and therefore the images differ because of marks that arose from wear and tear. Raw images such as those shown in Fig. 1 are saved with resolution of 300 dpi, RGB and tagged image file format (.tiff).

Twenty-eight distinct individuals contributed the 150 pairs of shoes in the database. Some individuals contributed more than 10 pairs of shoes. For each pair of shoes, we recorded information such as brand, model and size, but the model name was not always known with certainty.

**Fig. 2:**
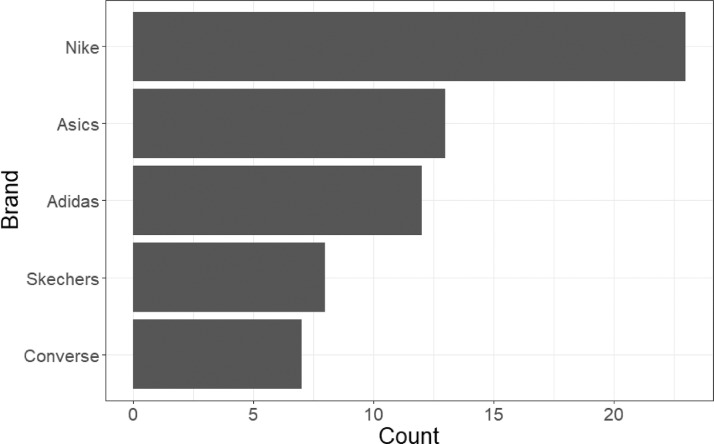
Observed frequencies of the five most popular brands among the 150 pairs of shoes. Nike is the most common brand in the collection of images.

**Fig. 3:**
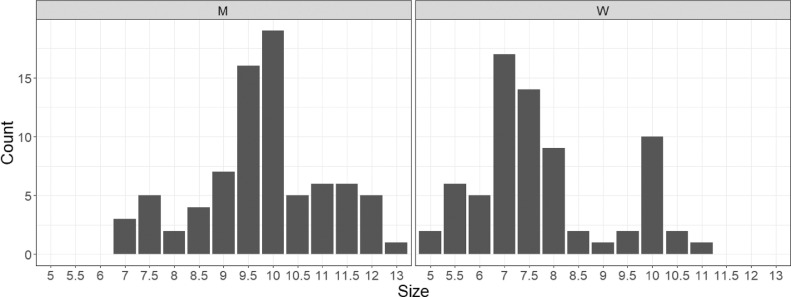
Histograms of shoe sizes among men (left panel) and women (right panel). For men, the most frequently observed sizes are 9.5 and 10. For women, size of 7 and 7.5 are the most popular sizes.

Figs. 2 and 3 display the frequencies of brands and shoe sizes in the database. From Fig. 2, we see that shoes of brand Nike, Asics, Adidas, Skechers and Converse are the most popular, at least among the 28 individuals who provided shoes for the data collection. All other brands are represented fewer than twice in the database. In terms of size, we distinguish between women and men sizes. Women's shoes ranged in size between 5 and 11, with more popular sizes being 7, 7.5 and 8. Size 10 is also popular, but this is because the individual who contributed the most shoes to the data collection was a female with that shoe size. In the case of men, sizes ranged from 7 to 13. The most frequently observed sizes were 9.5 and 10. Summary statistics and figures were obtained using R ([1]). The information about attributes of the collection of shoes (including the gender of the shoe owner) is available in the dataset.

## 2 Experimental design, materials, and methods

### 2.1 Experimental design

To build the collection of shoes, we asked colleagues at Iowa State University to bring in their used shoes. Therefore, the sample of shoes is a sample of convenience, and is not meant to be representative of any specific population of shoes. By accident, the database includes several pairs of shoes of the same model, brand and size owned by the same person. It also includes shoes of the same model, brand and size but owned by different individuals. Further, several pairs of shoes of the same model and brand but different size are also included. Mostly, the images represent a variety of shoe models with different outsole designs.

As mentioned above, the 300 shoes in the collection were scanned five times each. Thus, for a pair of shoes, there are 10 images, five for each shoe in the pair. In all, the database consists of 1500 images of shoe outsole impressions from 150 pairs of shoes.

The image files are labeled using the format AAA_BB_L/R_CC, where:

• AAA is a three-digit number between 001 and 028 that identifies the owner of the pair of shoes,
• BB is a two-digit number between 01 and 10 that identifies each pair of shoes belonging to the same individual,
• L/R denotes Left and Right and indicates whether the image corresponds to the left or the right shoe in a pair, and
• CC is a two-digit number between 01 and 05 that identifies the replicate image number for a shoe.

For example, the file labelled 015_01_L_01 is the image of the first replicate of the left shoe in the first pair owned by individual 15.

The database contains multiple images from the same shoe, from multiple shoes of the same make and model belonging to different individuals, and from multiple shoes of the same make and model belonging to the same individual. This enables investigation of the variability in 2D image attributes within shoe and owner, between shoes from the same owner, and between shoes from different owners.

### 2.2 Methods

**Fig. 4:**
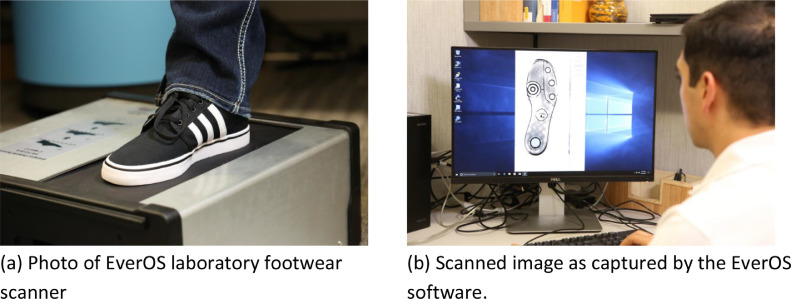
Participant stepping on the EverOS laboratory footwear scanner (left panel). Scanned outsole image (right panel).

The outsole scanner used in this data collection is an EverOS laboratory footwear scanner ([2], [3], [4]), shown in Fig. 4a. This scanner acquires the footwear outsole impression by detecting the weight of the wearer. It images the portions of the outsole that make contact with the scanning surface. To scan the shoes, we first clean the outsole to remove some dirt. Then, the person who is wearing the shoe steps on the surface of the scanner, as in Fig. 4a. As she steps on the scanner, the wearer shifts her weight from the heel to the toe so that the scanner captures the outsole pattern in detail. Once the scanner detects the outsole patterns, the image with the scanned outsole can be visualized on the screen in the special software provided by EverOS as in Fig. 4b.

The scanner saves the 2D outsole image in various different formats. In particular, images can be saved in the form of a tagged image file format (.tiff) and a joint photographic experts group (.jpg) format. We choose to save the scanned images as the format of .tiff with resolution of 300 dpi. The image is in RGB scale; the ruler recorded on the border of the image is shown in full RGB and the scanned outsole pattern is stored in greyscale.

## Appendix Supplementary materials

Image, application 1
